# Dietary Patterns, Carbohydrates, and Age-Related Eye Diseases

**DOI:** 10.3390/nu12092862

**Published:** 2020-09-18

**Authors:** Sarah G. Francisco, Kelsey M. Smith, Gemma Aragonès, Elizabeth A. Whitcomb, Jasper Weinberg, Xuedi Wang, Eloy Bejarano, Allen Taylor, Sheldon Rowan

**Affiliations:** 1 JM-USDA Human Nutrition Research Center on Aging, Tufts University, Boston, MA 02111, USA; sarah.francisco@tufts.edu (S.G.F.); kelsey.smith@tufts.edu (K.M.S.); gemma.aragones@tufts.edu (G.A.); elizabeth.whitcomb@tufts.edu (E.A.W.); jasper.weinberg@tufts.edu (J.W.); xuedi.wang@tufts.edu (X.W.); 2 Friedman School of Nutrition and Science Policy, Tufts University, Boston, MA 02111, USA; 3 Department of Ophthalmology, Tufts University School of Medicine, Boston, MA 02111, USA

**Keywords:** age-related macular degeneration, cataract, diabetic retinopathy, glaucoma, dietary pattern, Mediterranean diet, glycemic index, caloric restriction, intermittent fasting

## Abstract

Over a third of older adults in the U.S. experience significant vision loss, which decreases independence and is a biomarker of decreased health span. As the global aging population is expanding, it is imperative to uncover strategies to increase health span and reduce the economic burden of this age-related disease. While there are some treatments available for age-related vision loss, such as surgical removal of cataracts, many causes of vision loss, such as dry age-related macular degeneration (AMD), remain poorly understood and no treatments are currently available. Therefore, it is necessary to better understand the factors that contribute to disease progression for age-related vision loss and to uncover methods for disease prevention. One such factor is the effect of diet on ocular diseases. There are many reviews regarding micronutrients and their effect on eye health. Here, we discuss the impact of dietary patterns on the incidence and progression of age-related eye diseases, namely AMD, cataracts, diabetic retinopathy, and glaucoma. Then, we focus on the specific role of dietary carbohydrates, first by outlining the physiological effects of carbohydrates on the body and then how these changes translate into eye and age-related ocular diseases. Finally, we discuss future directions of nutrition research as it relates to aging and vision loss, with a discussion of caloric restriction, intermittent fasting, drug interventions, and emerging randomized clinical trials. This is a rich field with the capacity to improve life quality for millions of people so they may live with clear vision for longer and avoid the high cost of vision-saving surgeries.

## 1. Introduction

Vision loss is a pervasive health impairment affecting over 400 million people worldwide. Aging is a significant risk factor for several loss-of-vision eye diseases. As the global elderly population is growing, it is incumbent upon researchers to uncover mechanisms of ocular disease progression and identify strategies to prevent or slow these diseases. The four major age-related eye diseases are age-related macular degeneration (AMD), cataracts, diabetic retinopathy (DR), and glaucoma.

AMD is the leading cause of vision loss in industrialized countries with an estimated 196 million people between the ages of 30–97 affected by AMD around the world [1]. Broadly, AMD involves damage to the macula, an area of the retina necessary for high-acuity vision, and can lead to loss of central vision. AMD comes in two forms, “dry” AMD and “wet” AMD, with dry AMD accounting for the majority of cases. Dry AMD results from damage to the supporting cells of the retina called retinal pigmented epithelial cells (RPE) and is associated with a buildup of extracellular protein- and lipid-containing deposits called drusen. Wet AMD, while less common, is far more damaging as it is the result of aberrant angiogenesis with an increase in leaky blood vessels releasing fluid under the macula resulting in photoreceptor degeneration.

Cataract is the second most pervasive cause of age-related vision loss accounting for 51% of world blindness [2]. Cataract is the result of opacification in the lens of the eye that blurs and eventually obscures vision and is most commonly corrected with surgical removal of the cataract.

Diabetic retinopathy (DR) is the cause of vision impairment for an estimated 146 million people worldwide [3]. DR is a diabetic complication of the eye that is similar to wet AMD in that it involves damaged blood vessels in the retina that can cause blurred vision, floating spots, or blindness. High blood sugar as a result of diabetes can lead to damaged blood vessels in the inner retina, triggering these symptoms.

Glaucoma, the fourth major age-related eye disease, affects an estimated 76 million people across the globe. The mechanism(s) of disease progression of glaucoma are not well understood, but the result is a loss of peripheral vision from damage to the optic nerve and a measurable increase in intraocular pressure. Glaucoma is treated with drug therapy in the form of eye drops that reduce eye pressure, or laser treatment; however, these treatments cannot reverse or cure the disease.

Nutritional epidemiology has provided insights into the major age-related eye diseases, largely through observational studies, as well as some seminal randomized clinical trials (RCTs), most notably the Age Related Eye Disease Study (AREDS)1 and AREDS2 studies [4]. Multiple reviews indicate effects of individual food components through supplementation with whole foods, micronutrients, or macronutrients. AMD onset and progression are associated with low levels of carotenoids, antioxidant vitamins, and omega-3 fatty acids, and reduced intake of fruit, vegetables, and fish [5]. The AREDS studies demonstrated that high intake of vitamin A, vitamin, C, zinc, copper, and carotenoids could reduce the progression of AMD by approximately 25%. Age-related cataract has also been associated with vitamin and carotenoid status [6,7]. Although nutrients such as vitamins A, C, and E, lutein, zeaxanthin, and β-carotene were associated with reduced cataract risk in cohort studies, inconsistent results are reported in RCTs [8]. Observational cohort studies suggest that regular consumption of nitrate-rich leafy green vegetables is associated with reduced risk of glaucoma development [9]. There is also evidence that vitamins A and C are protective against glaucoma [10].

In this review, we evaluated the risk for age-related eye diseases from the perspective of dietary patterns, which take into account the effects of groups of various foods. Given the complexity of the nutrient interactions within dietary patterns, we anticipated that the evaluation of differences in dietary patterns will better inform about vision maintenance and health than supplementation with individual nutrients or single whole foods alone and could provide more effective dietary recommendations in the future.

## 2. Dietary Patterns and Eye Disease

Traditionally, dietary patterns have been divided into prudent dietary patterns and western dietary patterns. These can be defined based on a food factor analysis from dietary survey data, using techniques like principal component analysis to determine correlations between foods that are eaten together, or can be based on scores of adherence to pre-specified dietary patterns. These methodologies have been used to evaluate the major age-related eye diseases: AMD, cataract, glaucoma, and DR. For example, defined food intake patterns including prudent dietary patterns, the healthy eating index, and the Mediterranean diet have been related to risk for prevalence or progression of eye diseases in multiple cohorts. However, small differences in methodology likely contribute to some variation in findings using these metrics.

**Figure 1:**
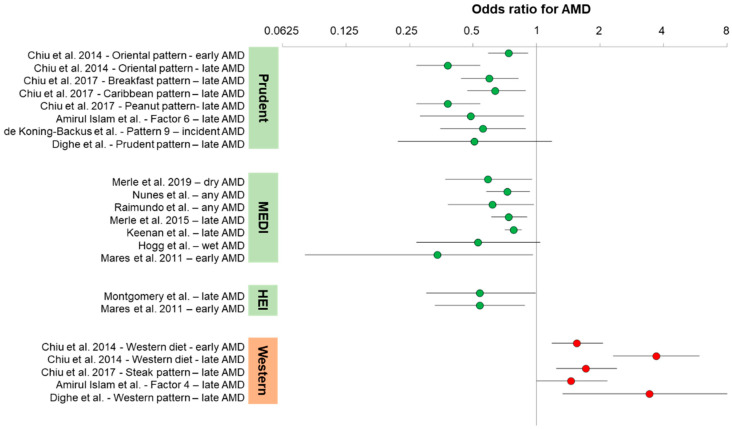
Odds ratios or relative risk ratios for age-related macular degeneration (AMD) from studies described in text (Section 2.1, Section 2.2, Section 2.3, Section 2.4).

Cohort designs are discussed briefly alongside findings. In general, prospective cohorts have the least biased design, while case–control studies have the most. In all designs, dietary pattern studies have the common limitations that the defined pattern is not necessarily the one that the individual consumed throughout their relevant dietary histories. Human dietary intake is extremely complicated and cannot be determined through food-frequency questionnaires alone. There are also gene–nutrient interactions. Some studies have a sufficient number of genotyped individuals to determine if gene–diet interactions exist between dietary patterns for age-related ocular diseases. As an example, some studies discussed below evaluated the major gene variants associated with AMD, Complement Factor H (CFH), and ARMS2 [11]. CFH genotypes include alleles associated with highly increased risk for AMD, as well as alleles associated with lower risk for AMD. Figure 1 shows a graphical representation of the odds ratios presented in the text for AMD. The odds ratios below are presented as age-adjusted and/or sex-adjusted, but not fully multivariate adjusted. Non-significant associations have been largely omitted for clarity.

### 2.1. Prudent Dietary Patterns and AMD

One of the first studies to evaluate association of AMD with dietary patterns was a factor analysis study of the American clinical AREDS cohort. Chiu et al. defined two major American dietary patterns and eight minor American dietary patterns. They referred to the prudent dietary pattern as the Oriental diet pattern, which consisted of the following food groups in order of importance: dark-yellow vegetables, cruciferous vegetables, green leafy vegetables, legumes, fruits, other vegetables, whole grains, tomatoes, fish and other seafood, rice, poultry, soup, and low-fat dairy products. Adherence to the Oriental diet pattern was associated with decreased risk of early AMD (OR = 0.74 (95% CI: 0.59–0.91); *P*_trend_ = 0.01) and late AMD (OR = 0.38 (95% CI: 0.27–0.54); *P*_trend_ < 0.0001) [12]. Three additional minor dietary patterns that partially overlapped with the Oriental dietary pattern were associated with decreased risk of advanced AMD: breakfast pattern (OR = 0.60 (95% CI: 0.44–0.82); *P*_trend_ = 0.004) characterized by cold breakfast cereals, fruit juices, whole grains, and fruit; Caribbean dietary pattern (OR = 0.64 (95% CI: 0.47–0.89); *P*_trend_ = 0.009) characterized by organ meats, poultry, fish and seafood, rice, and low-fat dairy; and peanut pattern (OR = 0.64 (95% CI: 0.46–0.89); *P*_trend_ = 0.03) characterized by peanuts, snacks, high-fat dairy, sweets, and desserts [13].

A similar approach was taken by the Melbourne Collaborative Cohort study, a longitudinal cohort primarily focused on evaluating diet and lifestyle in cancer prevention. Amirul Islam et al. identified six dietary patterns associated with early or late AMD [14]. They found that one pattern (factor 6), characterized by grains, fish, steamed or boiled chicken, vegetables, and nuts protected against late AMD (OR = 0.49 (95% CI: 0.28–0.87); *P*_trend_ = 0.008). Dietary patterns that consisted of fruits, vegetables, or salads did not have statistically significant associations with early or late AMD; there were no dietary patterns with significant associations with early AMD.

The Rotterdam Eye study was a prospective population cohort from the Netherlands. Using baseline dietary data, de Koning-Backus et al. defined nine dietary patterns from within a prudent diet and associated each with incident AMD. They found that one of those patterns (pattern 9) was associated with protection against incident AMD (OR = 0.56 (95% CI: 0.35–0.89)). This pattern included consuming vegetables (≥200 g/day), fruits (2×/day), and fish (2×/week) [15]. Surprisingly, other dietary patterns that included vegetables, fruits, and fish, but were constrained by intake levels of other foods like eggs, poultry, meats, or potatoes, did not reach statistical significance, suggesting that there may be negative off-sets from certain foods associated with a western dietary pattern.

Not all prudent dietary patterns have shown protective associations against AMD. A smaller study of the Irish Nun Eye Study identified a healthy dietary pattern containing fruits, vegetables, oily fish, nuts, and several other foods, but did not identify associations with AMD for any of their dietary patterns [16]. Notably, the healthy dietary pattern also included red meat and pizza, suggesting that, like in the Rotterdam Eye study dietary pattern analysis, protection from AMD may have as much to do with what is not consumed, as what is consumed. A prospective study from the ARIC (Atherosclerosis Risk in Communities) cohort identified a prudent dietary pattern and although the prudent dietary pattern showed a protective effect on late AMD, similar in magnitude to the studies above, it did not reach statistical significance (OR = 0.51 (95% CI: 0.22–1.18); *P*_trend_ = 0.054) [17].

### 2.2. Mediterranean Diet and AMD

The Mediterranean diet (MeDi) has been one of the most extensively studied dietary patterns and has been linked to improved health span, weight maintenance, and reduced chronic disease [18,19]. The MeDi resembles the prudent dietary patterns described above, but has additional emphasis placed on regular consumption of olive oil, nuts, legumes, and fish and seafood. Several different approaches to assessing adherence to MeDi exist, including a score called the alternative MeDi score. All are based on extensive dietary questionnaire data. It can be difficult to directly compare studies that use different methodologies because of differences in how MeDi adherences are determined. Nevertheless, it is impressive that, to date, MeDi adherence has been consistently associated with protection against AMD in different geographic cohorts that have very different baseline dietary preferences.

Two prospective European cohort studies, the Rotterdam study from the Netherlands and the Alienor study from France, termed the EYE-RISK consortium, have evaluated associations between MeDi scores and AMD. Merle et al. reported that in both cohorts individually and combined, high adherence to MeDi, as assessed by the 9-point European-based MeDi score (mediSCORE > 6), was associated with reduced incidence of dry AMD (OR = 0.59 (95% CI: 0.37–0.95); *P*_trend_ = 0.04) [20]. They evaluated the type of advanced AMD separately, and only dry AMD maintained a significant protective association (OR = 0.42 (95% CI: 0.2–0.9); *P*_trend_ = 0.04). The association with wet AMD was of a similar magnitude, but did not reach statistical significance, perhaps due to a smaller number of cases. No gene–diet interaction between MeDi and the CFH risk allele (Y402H: rs1061170) was observed.

Case–control studies have been widely used to evaluate MeDi scores and AMD. The Coimbra Eye Study evaluated the association between MeDi adherence and risk of AMD in two Portuguese populations using the 9-item mediSCORE [21] and found that mediSCOREs > 6 were associated with protection from AMD (OR = 0.73 (95% CI: 0.58–0.93)). A nested subset of the Coimbra Eye Study was similarly analyzed and showed a very similar protection for any AMD (OR = 0.62 (95% CI: 0.38–0.97)) [22].

An American clinical cohort consisting of patients from the AREDS1 and AREDS2 trials has also been scored for association of MeDi with advanced AMD using the alternative MeDi score (aMeDi), designed for U.S. populations. These studies demonstrated that MeDi not only protects against late AMD, but also that it can interact with a particular risk allele of the CFH alternative complement inhibitor (Y402H: rs1061170). Merle et al. showed that aMeDi scores > 6 were associated with protection from advanced AMD (OR = 0.74 (95% CI: 0.61–0.90), *P*_trend_ = 0.005) [23]. They also described a gene–diet interaction between the non-risk allele of CFH (Y402) and aMeDi score > 6 (*P* = 0.04), wherein individuals with two copies of the risk allele of CFH (Y420H) were not protected from advanced AMD by high adherence to MeDi. These findings were expanded recently by Keenan et al., who used aMeDi scores to evaluate the combined AREDS1 and AREDS2 cohorts [24]. This study replicated the protective effect of MeDi on late AMD (OR = 0.78 (95% CI: 0.71–0.85); *P*_trend_ < 0.0001), and further defined enhanced protection against dry forms of AMD compared with wet forms of AMD. In particular, individuals within the AREDS2 cohort did not show a significant protective effect of high aMeDi scores on wet AMD. Keenan et al. replicated the interaction between a CFH risk allele and MeDi and expanded these findings by evaluating separately a protective allele of CFH (rs10922109) and found a gene–diet interaction (*P* = 0.01), wherein only individuals containing at least one protective CFH allele were protected against advanced AMD by MeDi. The Keenan et al. study, as well as a follow-up study from Merle et al., found that MeDi was associated with delayed progression of drusen to large drusen size, indicating that one protective impact of MeDi may be mediated directly in the eye on drusenogenesis [24,25].

Other cohorts have evaluated advanced AMD relationships to MeDi. In the European Eye Study, Hogg et al. found that adherence to MeDi, as measured using a mediSCORE > 6 similar to the Coimbra studies, led to reduced wet AMD (OR = 0.53 (95% CI: 0.27–1.04); *P*_trend_ = 0.01), without an interaction with the CFH risk allele (Y402H: rs1061170) (*P* = 0.89) [26]. No significant associations were found for early AMD. Hogg et al. also reported a weak trend between MeDi and large drusen (*P* = 0.05). It is curious that the European cohorts have not found interactions between MeDi and the CFH risk allele, as reported in the U.S. AREDS study. One possible explanation is that the AREDS study already enrolled patients with intermediate AMD and might have had an overabundance of the CFH risk allele. A second possibility suggested by Keenan et al. is that the relevant allele is the CFH protective allele, which was likely present in those lacking the CFH risk allele [24]. A gene–diet interaction between the risk allele of CFH and fish intake, a critical protective component of MeDi, may explain the MeDi–CFH genetic interactions [11,24].

While the associations between MeDi and late AMD have been well-replicated, the relationship between MeDi and early AMD is less clear. In an analysis of the CAREDS (Carotenoids in Age-related Eye Disease Study) cohort, using the aMeDi score cutoffs, Mares et al. reported a significant protective effect of MeDi on early AMD (OR = 0.34 (95% CI: 0.08–0.96); *P*_trend_ = 0.046), but noted that only 53 women in their cohort fit the 6-9 aMeDi score cutoff [27]. Determining whether MeDi can prevent incident AMD will likely require several new cohort studies from different geographical regions, and potentially different, geographically relevant scoring systems for MeDi.

### 2.3. Healthy Eating Index and AMD

Many western populations show overall poor adherence to MeDi, but there are alternative defined dietary patterns that have been used to assess dietary relationships to health. One of those is the healthy eating index (HEI). Two studies have evaluated associations between early or late AMD and HEI scores. Montgomery et al. used the alternative HEI to assess its relationship to advanced AMD in a case–control study and found that it was protective (OR = 0.54 (95% CI: 0.30–0.99)) [28]. They also used the traditional HEI and found a protective effect that did not achieve statistical significance, possibly because of the relatively small size of the cohort (666 total). Aspects of the alternative HEI that may track better with AMD protection include components for trans-fat intake and a ratio of polyunsaturated fats to saturated fats. Mares et al. also used a modified HEI to examine relationships to early AMD, as they did above for MeDi, and found a protective association (OR = 0.54 (95% CI: 0.33–0.88), *P*_trend_ = 0.01) [27]. These protective associations were also true when analyzed for large drusen, pigmentary abnormalities, or total AMD including late AMD.

### 2.4. Western Diet and AMD

The flipside of the healthy dietary patterns presented above as prudent dietary patterns, MeDis, or HEI is the western diet. Although categorized differently based on food frequency questionnaire data, the common features of western dietary pattern are higher intakes of red meat, saturated fats, highly processed foods, sweets and desserts, and sugar-sweetened beverages. Chiu et al. characterized such a western dietary pattern and showed a statistically significant association with early AMD (OR = 1.56 (95% CI: 1.18–2.06); *P*_trend_ = 0.01) and late AMD (OR = 3.7 (95% CI: 2.31–5.92); *P*_trend_ < 0.0001) [12]. Chiu et al. separately identified a minor dietary pattern, overlapping with the western dietary pattern, coined the steak pattern, with an emphasis on red meat, potatoes, gravies, and butter or margarine, that was associated with late AMD risk (OR = 1.73 (95% CI: 1.24–2.41); *P*_trend_ = 0.02) [13].

Using a similar factor analysis approach to dietary patterns, Amirul Islam et al. characterized a red meat dietary pattern (Factor 4) that also included processed fish, eggs, and low intake of whole wheat or rye bread [14]. The red meat dietary pattern was associated with increased risk for late, but not early, AMD (OR = 1.46 (95% CI: 1.0–2.17). Their factor analysis also identified a separate subset of the typical western dietary pattern containing mostly processed foods (Factor 5) that did not associate with any AMD. However, Factor 5 also included foods like peanuts, tea, and dairy that have been associated with protection from AMD, suggesting that potentially harmful foods in the dietary pattern could be counterbalanced by the intake of beneficial foods.

As described above, prospective studies are powerful designs for dietary pattern analysis. Dighe et al., using the ARIC cohort, evaluated dietary patterns with risk for AMD among 1278 individuals, and identified major prudent and western dietary patterns [17]. Individuals that adhered to a western diet were at increased risk for advanced, but not early, AMD (OR = 3.44 (95% CI: 1.33–8.87); *P*_trend_ = 0.014). The findings from Dighe et al. and Amirul Islam et al. that the Western diet patterns were only associated with late, but not early, AMD seem to be at odds with the Chiu et al. findings. In the AREDS cohort, individuals were enrolled based on having intermediate AMD present in at least one eye; therefore, the individuals in this study may be at increased risk of early AMD in the other eye. Prospective studies using healthy individuals at baseline are therefore better suited for the evaluation of early AMD.

### 2.5. Cataract and Dietary Patterns

Age-related cataract is the most prevalent condition associated with vision impairment and blindness in older adults worldwide [29]. Although it can be treated surgically, this is cost-prohibitive in many developing nations, where there are insufficient numbers of surgeons to meet the challenge. Prevention through lifestyle intervention may be the only viable treatment option. It is therefore important to determine whether dietary patterns impact cataract prevalence or progression. A role for nutrition in cataract formation has been widely assumed based on findings that micronutrient status is strongly associated with cataract risk (reviewed in [6,8]). However, supplementation studies, most of which were short term, have largely failed, suggesting that either longer term intervention trials are required and/or multiple dietary components may be involved in cataract formation. Micronutrient or vitamin status may be acting as a surrogate for diet quality.

Given the emphasis on phytochemical-rich foods in MeDi, it is hypothesized that adherence to MeDi might delay cataractogenesis. Garcia-Layana evaluated the incidence of cataract surgery from the landmark PREDIMED (Prevención con Dieta Mediterránea) clinical study, where 5802 men and women were randomized to a MeDi supplemented with extra-virgin olive oil, a MeDi supplemented with mixed nuts, or a low-fat control diet that conformed to the American Heart Association (7272 Greenville Avenue, Dallas, Texas) guidelines [30]. There was no difference in the incidence of cataract surgery (a surrogate for advanced cataract formation) in any of the diet groups. Nevertheless, since cataracts can be present for long periods of time before surgical removal is necessitated, the study was not designed to test whether MeDi could affect earlier steps in cataractogenesis. Further, the control arm was a different kind of healthy diet. A comparison against a western diet in a prospective cohort might still reveal important roles for MeDi in cataract prevention or a role for western diet in cataract promotion.

The HEI has also been evaluated as a potentially beneficial dietary pattern for cataract prevention. Moeller et al. evaluated HEI scores in a subset of the Nurses’ Health Study prospective cohort study [31]. They found that adherence to the HEI was associated with reduced prevalence of nuclear cataract (OR = 0.44 (95% CI: 0.26–0.76); *P*_trend_ = 0.001). Interestingly, another indicator of dietary pattern at that time, the recommended food score (RFS) did not show any relationship to nuclear cataract. The authors pointed out that the RFS correlated better with dietary variety than dietary pattern.

Mares et al., using hybrid cross-sectional and prospective data from the CAREDS prospective study and the WHI (Women’s Health Initiative) observational study, evaluated associations between nuclear cataract and two versions of the HEI, HEI-1995 and HEI-2005 [32]. They found that adherence to HEI-1995 was protective for nuclear cataract (OR = 0.57 (95% CI: 0.4–0.81); *P*_trend_ = 0.01), whereas adherence to the HEI-2005 was not. This discrepancy was resolved by the finding that the highest quintile of adherence to HEI-2005 also had the highest intake of oils, an independent co-variate for nuclear cataract.

A prospective cohort study evaluated an Australian HEI to determine whether adherence to healthy dietary patterns would affect the incidence of nuclear, cortical, or posterior subcapsular cataract within the Blue Mountains Eye Study [33]. Tan et al. found marginally non-significant decreased risk of incident nuclear cataract associated with each unit increase in total HEI score (OR = 0.95 (95% CI: 0.87–1.01); *P*_trend_ = 0.08). No association was evident between increased total HEI score and incident cortical or posterior subcapsular cataract.

Smaller studies have also suggested the protective effects of adherence to HEIs on cataracts. Ghanavati et al. utilized a case–control study in Iran and assessed the association of cataract with HEI [34]. They found that all categories of HEI were protective against cataract except for the lowest quartile (OR = 0.19 (95% CI: 0.09–0.4); *P*_trend_ < 0.01). These data speak more to the detrimental effect of a poor diet on cataractogenesis than protection from healthy dietary pattern. Indeed, a follow-up study performing factor analysis on the dietary data to extract nutrient patterns identified two unhealthy nutrient patterns, one termed a sodium pattern that also contained high amounts of carbohydrates and proteins (OR = 1.97 (95% CI: 1.09–3.96)), and the other termed a fatty acid pattern that included trans fats, and thus a surrogate for processed meats and foods (OR = 1.94 (95%CI: 1.1–3.86)) [35].

### 2.6. Glaucoma and Dietary Patterns

The predominant risk factor for glaucoma is age, and there is not yet a well-established nutrient or food association with glaucoma and a very small number of studies have rigorously tested dietary patterns as risk factors for glaucoma. A systematic review found that some micronutrients like selenium and iron may be associated with increased risk for glaucoma, while components of dark-green leafy vegetables, specifically glutathione, flavonoids, and nitric oxide, were significantly associated with decreased risk for glaucoma [36]. The disparate effects of nutrients that synergize within a given dietary pattern may explain the lack of clear associations between dietary patterns and glaucoma.

### 2.7. Diabetic Retinopathy and Dietary Patterns

Diabetic retinopathy (DR) is a microvascular complication of diabetes that is associated with increased age, increased duration of diabetes, and worsened control of hyperglycemia. A large body of literature has concerned itself with the relationships between nutrients, diet, and dietary patterns and control of diabetes [37]. Treatments that improve diabetes should lower the progression to DR. Nevertheless, a relatively few studies have tested whether adherence to healthy or unhealthy dietary patterns alter the incidence of DR, as assessed ophthalmologically.

Adherence to MeDi has been shown to prevent diabetes, as evaluated within the PREDIMED randomized clinical trial [38]. It was thus logical to determine whether DR might be similarly prevented. In a post-hoc analysis of the PREDIMED trial, Díaz-López et al. assessed whether individuals that consumed a MeDi containing olive oil or nuts were protected from developing DR [39]. The analysis was limited to individuals with type 2 diabetes. Comparing MeDi with the control diet, there was a significant reduction in incident DR (OR = 0.59 (95% CI: 0.37–0.95)). A further comparison of the MeDi supplemented with extra-virgin olive oil had a similar protective effect on DR (OR = 0.57 (95% CI: 0.33–0.98)), while the MeDi supplemented with nuts had a non-significant risk reduction (OR = 0.62 (95% CI: 0.34–1.11)) for DR. As this study was a post-hoc analysis of a randomized clinical trial, future studies are required that evaluate incident DR from a prospective cohort to confirm these findings.

## 3. Physiological Effects of Dietary Carbohydrates

Each of the dietary patterns outlined above includes varying ratios of macronutrients from whole food sources. Although exploring associations of disease with dietary patterns rather than individual nutrients may better reflect the effects of the human diet, it may also still be useful to apply a reductionist approach and evaluate the effect of individual nutrients on the physiology and mechanisms of pathogenesis. Carbohydrates vary greatly in structure and function and have wide-ranging physiological effects outside of the eye. Here, we discuss the effects of different carbohydrates on the body and how these changes lead to downstream effects in the eye. Given that the eye is distal from the sites of digestion and absorption of carbohydrates, contextualizing the eye effects with mediating factors, such as gut microbiome changes and inflammation, can inform our thinking regarding eye health outcomes.

### 3.1. Physiological Effects of Hyperglycemia

The physiological effects of dietary carbohydrates are dependent upon the structure and composition of the carbohydrate as well as host characteristics including gut microbiome composition and genetics. The structure of the carbohydrate determines the rate of monosaccharide release and absorption. Simple sugars and highly branched starches, such as those found in refined flour, are rapidly digested and absorbed, resulting in a quick and substantial increase in blood glucose concentration, mirrored by a rise in insulin [40]. Frequent consumption of rapidly digested carbohydrates can lead to insulin resistance and eventual exhaustion of the pancreas’ ability to maintain glucose homeostasis, leading to chronically elevated blood glucose, which has far ranging effects including increased gut permeability [41], chronic systemic inflammation and oxidative stress [42], and increased protein damage due to advanced glycation end products (AGEs) [43,44], which have been associated with many diseases including retinopathy.

The eye is particularly vulnerable to damage from direct and indirect effects of chronically elevated blood glucose due in part to its limited capacity for cellular turnover, limited roles for glucose transporters, and the high metabolic activity of the retina. Hyperglycemia has been clearly associated with DR, AMD, and cataracts [45]. There are multiple co-occurring pathways by which hyperglycemia can lead specifically to eye disease. Glucose entry into the retina is mediated by glucose transporter 1 via facilitated diffusion, and elevated blood glucose leads to increased cellular glucose in the eye [46]. This increase in cellular glucose can result in cell damage and apoptosis via an accumulation of AGEs [47], increased metabolism leading to reactive oxygen species accumulation and decreased glutathione, and protein kinase C activation leading to immune hyperactivity [45]. Additionally, aberrant vasculature present in retinopathy can be induced by AGEs and protein kinase C activation [45]. Finally, the effects of hyperglycemia on eye health may be mediated by systemic health conditions including dyslipidemia, which is associated with the formation of deleterious lipid droplets in the eye [48], as well as chronic low-grade inflammation, which triggers immune hyperactivity in retinal cells [49].

### 3.2. Dietary Fiber, Gut Microbiome, and Inflammation

Replacing rapidly digested starch with resistant starches, which humans have limited capacity to digest, has been shown to attenuate the rise in blood glucose following the meal and prevent the long-term negative health outcomes of a high carbohydrate diet [50]. Research from our group has found that replacing a rapidly digested starch with resistant starch can arrest or reverse the development of retinal lesions and photoreceptor layer thinning in the eyes of aged mice [44,51,52].

As the rate of digestion of resistant starch can be slower than the rate of transit through the small intestine, a portion passes into the large intestine, leading this carbohydrate to be classified as a dietary fiber [53]. Dietary fibers are a diverse class of carbohydrates with wide-ranging physiological effects. By increasing bulk and viscosity, dietary fibers slow the digestion and absorption of nutrients such as monosaccharides and fatty acids and improve bowel movement regularity. Through decreased calorie absorption, increased dietary fiber can reduce and prevent obesity and associated disease [54].

Additionally, dietary fibers provide metabolic substrate for commensal bacteria in the colon, which then produce short-chain fatty acids (SCFAs). SCFAs have effects throughout the body, including improved gut epithelium integrity, decreased appetite, altered fatty acid synthesis, and decreased systemic inflammation [55]. Increasing the substrate available to carbohydrate-utilizing bacteria increases their ability to out-compete pathogens and bacteria that produce harmful metabolites such as trimethylamine [56], which has been linked to cardiovascular disease [57]. Finally, some dietary fibers, such as arabinoxylan found in whole wheat, contain potent antioxidants [58], which further alter the gut microbiome [56], reduce intestinal oxidative stress [58], and may reduce systemic oxidative stress [59], an established pathoetiologic mechanism for cardiometabolic disease and retinopathy.

In addition to the physiological effects of SCFAs, the gut microbiome can affect multiple pathways implicated in eye disease. Increased gut permeability, which can be a result of intestinal inflammation and/or hyperglycemia, allows the translocation of microbial products, which can bind receptors in the eye and signal ocular inflammation [60]. Additionally, microbial metabolites may cross the blood–retina barrier and act as signaling molecules in the eye. Multiple molecules produced in the gut, including serotonin, have been associated with eye disease severity [44]. Finally, the gut microbiome can indirectly affect eye health by affecting mediators such as insulin sensitivity and adiposity.

Dietary carbohydrate content and composition are major determinants of gut microbiome function [61]. Consequently, these factors are likely important mediators of the effects of dietary patterns on gut microbiome composition and downstream health outcomes. A western diet, characterized by low fiber intake and high simple sugar and starch content, results in increased gut permeability and microbial translocation, increased intestinal inflammation, and decreased resilience to enteric infections [62]. A Mediterranean diet, which includes increased consumption of produce, nuts, and legumes, promotes the proliferation of bacteria associated with several beneficial changes in gut microbiome function, including increased SCFA production and decreased secondary bile acids and inflammatory markers [63].

### 3.3. High Glycemic Diet and Age-Related Eye Diseases

As thoroughly reviewed previously, advanced glycation end products (AGEs), the result of non-enzymatic addition of sugars and their metabolites to proteins and their subsequent toxic accumulation, have been implicated in a wide variety of age-related diseases, including ocular diseases such as AMD, cataract, and DR [64,65,66]. These cytotoxic AGE accumulations occur in individuals with high blood glucose levels, including individuals with diabetes or pre-diabetes with chronic hyperglycemia. Additionally, high glycemic diets, whether measured by glycemic index or glycemic load are associated with eye disease progression, including AMD and cataract, as measured in case–control studies and epidemiological analyses [64,67,68,69,70,71]. A recent case–control study demonstrated a significant, direct relationship between daily glycemic index, glycemic load, and insulin load on cataract risk [67]. These studies highlight the importance of dietary carbohydrate source and structure in eye disease progression and prevention.

Many animal studies have been done to model eye disease progression in a high glycemic (HG) context and to recapitulate the ameliorative effects of a low glycemic (LG) diet. AMD-like features, namely, RPE hypopigmentation and atrophy, lipofuscin accumulation, and photoreceptor degradation, were observed in male C57BL/6J mice fed an HG diet [44]. These AMD features were not seen in the group that was fed an LG diet and importantly, the intervention with the LG diet from the starting HG-fed animals improved eye outcomes compared with HG-fed throughout, indicating that dietary intervention with changes in carbohydrate content directly affects eye outcomes.

The high glycemic diet models a western diet that is high in rapidly digested starch, but there are several other models of unhealthy western diets, including a high fat diet commonly used in obesity studies as well as an extreme western diet model using a high fat/high sugar diet. In a mouse model of glaucoma using induced intraocular pressure by injecting endotoxin-free saline, mice fed a high fat/high sucrose diet were more susceptible to damage to retinal ganglion cells under increased intraocular pressure [72]. The damage measured was used as a measure of glaucoma in these animals and this susceptibility in the high fat/high sucrose diet group was not improved with exercise, suggesting that this type of dietary change is severely detrimental and cannot be compensated by other aspects of a healthy lifestyle. The diet used in this study was an extreme model of unhealthy eating, the equivalent of consuming “fast food” with high fat content paired with high sugar, soda-like drinks. In the Nrf2 knockout mouse, a model of age-related macular degeneration, HG diet induced more severe AMD features that were ameliorated with LG diet, underscoring the strong effects of diet over a genetic factor in disease progression [51].

## 4. Future Directions for Nutrition and Age-Related Eye Diseases

Although nutrition research in the eye field has mainly focused on the contributions of macronutrients/micronutrients to eye health, there is an increasing interest in determining if other dietary parameters, such as how many or when calories are ingested, might impact the ocular function. Although not yet in clinical practice, a body of clinical evidence and data from animal models supports that dietary trends are potential approaches for the prevention and treatment of vision disorders.

### 4.1. Calorie Restriction and Ocular Diseases

Among the different interventions regarding dietary trends to slow down age-related functional decline, caloric restriction is the best studied strategy in the context of ocular diseases. Caloric restriction, which is defined by low intake of calories without undernutrition, has been shown to trigger anti-aging mechanisms by decreasing mTOR activity and IGF/insulin signaling and enhancing sirtuin activity [73]. The therapeutic benefit of caloric restriction has been explored in different eye-related disorders including cataract, AMD, dry eye disease, and corneal endothelial cell loss.

The best-documented caloric restriction-related studies in eye diseases are analyses of cataract onset and progression. Caloric restriction was shown to delay age-related cataract in different dark-eyed mouse and rat strains [74,75,76,77,78,79]. The molecular mechanism behind the protective effect mediated by caloric restriction remains unclear. Caloric restriction delays the age-related decline in proliferation capacity of lens epithelial cells [80,81], attenuates the shortening of telomeres [82], and lowers the aggregation of β- and γ-crystallins [83]. Caloric restriction’s protective benefit might be also due to enhanced tissue antioxidant capacity by retarding the age-related aggregation of crystallins [75,84]. However, the literature is inconclusive and lower levels of superoxide dismutase and no differences in primary antioxidants, such as ascorbate, were found in calorically restricted animals [76,78,79,84].

Caloric restriction also limits age-related dysfunction in retinal tissues. Caloric restriction attenuates inherent age-related loss of retinal ganglion cells and is also neuroprotective against ischemia [85,86]. Age-related photoreceptor cell death is attenuated in calorically restricted brown Norway rats [87,88]. In the neural retina of this rat strain, caloric restriction diminishes age-related protein insolubilization and blunts age-related decline in total soluble thiols [89,90]. However, the protective role of caloric restriction in photoreceptors is strain-dependent as it resulted in increased light-dependent photoreceptor cell death in the neural retina of Fischer 344 rats [87,88]. Caloric restriction also reduces the age-related accumulation of lipofuscin associated with RPE dysfunction in male Wistar rats [91]. In addition, although not statistically significant, calorically restricted rhesus monkeys had lower prevalence and severity of AMD-like ocular pathology [92].

### 4.2. Intermittent Fasting and Eye Diseases

In spite of data supporting a therapeutic role of caloric restriction on eye heath, the implementation of caloric nutrition as a disease prevention or treatment strategy is challenging due to its large impact on quality of life, especially in clinical settings, and recent research has sought ways to mimic the benefits of caloric restriction with less strict guidelines. One such strategy is intermittent fasting, which involves restricting food intake to specific periods of time followed by extended fasting. Alternate-day fasting for 6 months accelerated the recovery in inner retinal function following intraocular pressure challenge in C57BL/6J mice [93]. Additionally, every-other-day fasting showed a neuroprotective effect on glaucomatous pathology in EAAC1^−/−^ mice, a mouse model of normal tension glaucoma, suppressing retinal ganglion cells and retinal degeneration without altering intraocular pressure [94]. The mechanisms behind the beneficial properties of fasting remain unclear, but several reports suggest that inducing autophagy through fasting improves retinal ganglion cell survival in animals subjected to ischemia [95]. In addition, intermittent fasting restructured the gut microbiota toward species that modulate the production of neuroprotective bile acids, preventing diabetic retinopathy [96].

### 4.3. Drug Interventions and Eye Diseases

While several pharmaceutical options have been evaluated for efficacy against age-related eye diseases, here, we focus on those that are widely applied for other metabolic disorders, such as diabetes, that may elicit similar effects as dietary changes. Diabetes and hyperglycemia are risk factors for several of the age-related eye diseases outlined above. Similarly, cardiovascular disease (CVD) is frequently a comorbidity for age-related pathologies. Therefore, efforts are currently underway to establish whether drugs used to treat these conditions additionally have beneficial eye outcomes.

Drugs for the treatment of diabetes have the potential to attenuate age-related vision loss through reducing hyperglycemia. Metformin, a common treatment for type 2 diabetes, is associated with decreased AMD incidence [97,98]. Additionally, metformin has been detected in the aqueous humor of diabetic patients taking the drug, but the association of metformin use and cataracts has not been evaluated [99]. Finally, long-term regular use of high-dose metformin is associated with reduced risk for glaucoma and reduced severity of DR [100,101,102].

Statins are used to treat high cholesterol in patients at risk for CVD and are one of the most prevalent prescriptions, with 28% of adults over 40 taking statins in 2012 [103]. Many diabetic and pre-diabetic patients take statins and are at risk for different age-related eye diseases, but the association of statin use and AMD is inconclusive [104,105]. Information surrounding statin use and cataracts is also inconclusive: of three meta-analyses of observational and randomized controlled trials for statins and cataracts, one reported reduced risk for cataract, one reported a mild increased risk, and the third was inconclusive [106,107,108]. Similarly, a recent retraction of a study analyzing glaucoma and statin use revealed that there was no decreased risk of primary open-angle glaucoma with statin use over 5 years (OR = 0.93 (95% CI: 0.75–1.15); *P*_trend_ = 0.49) [109,110].

### 4.4. Randomized Controlled Clinical Trials

Observational studies are powerful but are limited by their inability to prove causation from association. RCTs are essential to move from research findings to clinical practice. The largest nutrition-based randomized controlled study in targeting age-related eye diseases was the AREDS study by the National Institutes of Health, which focused on micronutrient supplementation as an intervention strategy to analyze disease incidence and progression for AMD and cataracts [111]. While this study aimed to recommend supplements to prevent or slow AMD and cataract development, it is a model for future randomized controlled studies that may be done with macronutrient or dietary pattern recommendation as the previously described evidence suggests that making changes to these aspects of nutrition has more drastic effects on disease than micronutrient supplementation.

RCTs specifically investigating dietary patterns and age-related eye diseases have not yet been performed. Recently, the first study of this kind has been proposed for Australian patients with any form of AMD, with the intervention group to receive detailed dietary information as it relates to macular health as well as regular contact with a registered dietician, while the control group will receive generic nutrition advice [112]. Older adults with varying stages of AMD will be recruited, which will allow researchers to identify the most effective timing of dietary intervention to prevent disease progression. The recommendations given by the dietician will emphasize the consumption of vegetables, fruits, fish and nuts, or low glycemic index, all associated with improved eye outcomes [112].

There have also been several RCTs that explored the effect of dietary pattern on other health outcomes, and age-related eye diseases can be added as measured outcomes within these cohorts to analyze the long-term effects of diet on age-related vision loss in older adults. An example of such an approach is the post-hoc analysis of cataract surgery rates within the PREDIMED RCT, described above in Section 2.5 [30].

## 5. Strengths, Limitations, and Outlook

Here, we outline many epidemiological studies analyzing the correlation between various dietary patterns, macronutrient contributions to diet, and eye disease outcomes. These studies have highlighted the effect of diet on age-related eye diseases, identified important dietary components, and formed the basis for the animal models that have shaped our understanding of the interrelatedness between diet and eye health. However, RCTs of dietary patterns are the essential next step to further our progress toward translating evidence to practice and preventing and treating age-related vision loss. Future RCTs of dietary patterns need to be performed in older adults and measure specific ocular outcomes, including incidence and progression of AMD, DR, cataract, and glaucoma. An emphasis should be placed on the prevention of early disease, which would have greater potential to extend the health span.

Although we discussed the role of dietary carbohydrates on age-related eye diseases, there is also likely an important role for dietary lipids and lipid composition in these diseases. For example, there is evidence suggesting that omega-3 fatty acids are protective against wet AMD [113], but more work is required with regard to lipids and age-related eye outcomes.

## 6. Conclusions

**Figure 2:**
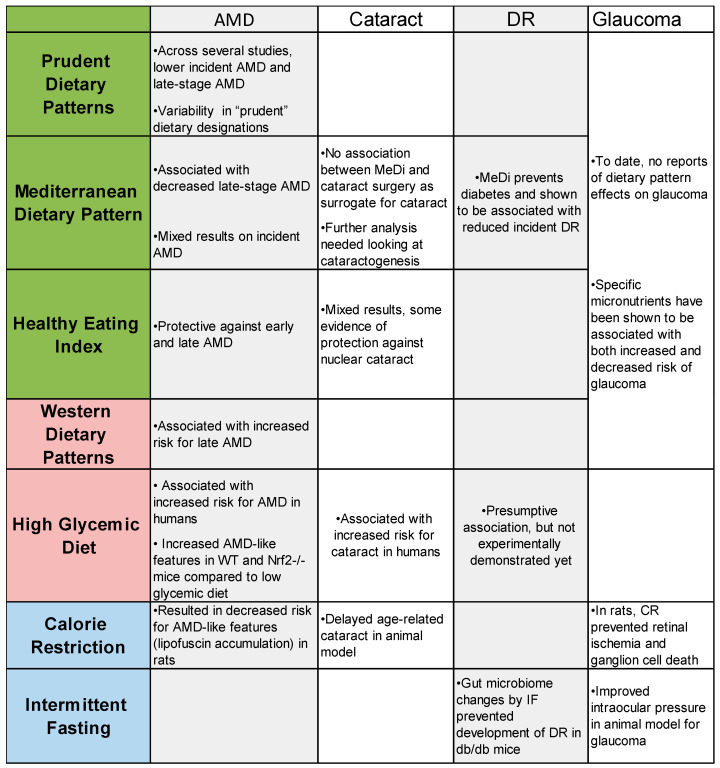
Summary of findings linking dietary patterns or select dietary interventions to age-related eye disease. Abbreviations: CR, caloric restriction; IF, intermittent fasting; DR: diabetic retinopathy.

A summary of the findings of this review are included in Figure 2. Based on our literature review of dietary patterns and age-related eye diseases, we found strong evidence about dietary patterns in regard to AMD and some in cataract, but there is surprisingly little conclusive evidence linking specific dietary patters with DR and glaucoma. Across studies looking at AMD progression, there are consensus findings that adherence to a prudent dietary pattern, the Mediterranean diet, and the healthy eating index all protect against AMD and that the western dietary pattern can accelerate AMD progression. In cataract, there is more evidence indicating that an unhealthy western dietary pattern accelerates cataractogenesis, but there is some evidence to suggest that dietary patterns such as the HEI may be protective against cataract progression. In order to best design future RCTs involving dietary pattern changes, animal models are important to uncovering the mechanisms that could explain human epidemiology. Animal models can then be used to implement whole dietary changes that are expected to benefit human participants in the future as exemplified here through mice fed a low or high glycemic diet. Information from animal studies can inform the future of nutrition and age-related eye disease research through RCTs that are specifically designed to analyze eye health outcomes.
